# Psychological stress and cardiovascular risk among women in Brazilian communities: a cross-sectional study

**DOI:** 10.1590/0102-311XEN234522

**Published:** 2024-07-29

**Authors:** Antônio José C. Mattos, Alvaro Avezum, João Italo D. França, Maria Cristina O. Izar, João Fernando M. Ferreira, Luciano Ferreira Drager, José Francisco K. Saraiva, Henrique Andrade R. Fonseca

**Affiliations:** 1 Sociedade de Cardiologia do Estado de São Paulo, São Paulo, Brasil.; 2 Hospital Alemão Oswaldo Cruz, São Paulo, Brasil.; 3 Instituto Dante Pazanesse, São Paulo, Brasil.; 4 Universidade Federal de São Paulo, São Paulo, Brasil.; 5 Universidade de São Paulo, São Paulo, Brasil.; 6 Pontifícia Universidade Católica de Campinas, Campinas, Brasil.; 7 Hospital Israelita Albert Einstein, São Paulo, Brasil.

**Keywords:** Psychological Stress, Cardiovascular Diseases, Women, Developing Countries, Estresse Psicológico, Doenças Cardiovasculares, Mulheres, Países em Desenvolvimento, Estrés Psicológico, Enfermedades Cardiovasculares, Mujeres, Países en Desarrollo

## Abstract

Psychosocial evaluations are rarely conducted with community-dwelling individuals, especially those with higher risk of cardiovascular disease. This study aims to evaluate the perceptual stress and cardiovascular risk among women in a large cross-sectional study performed in Brazilian communities. Subjects aged over 18 years were included out of 500 public basic health units (BHU) in Brazil. All subjects were subjected to a clinical consultation and questionnaires application. Data were used to identify healthy lifestyle, smoking status, and self-perception of psychological stress. The *National Health and Nutrition Examination Survey* (NHANES) risk score (NRS) was used to estimate cardiovascular risk. Ethnicity information was self-reported, considering white versus non-white (black, brown, and mixed-race) women. A total of 93,605 patients were recruited from a primary care setting, of which 62,200 (66.4%) were women. Intense and severe auto-perception of stress was higher within non-white women at home (p < 0.001), at work (p = 0.008), socially (p < 0.001), and financially (p < 0.001) compared to white women. Therefore, the NRS indicates that non-white women had higher cardiovascular risk, lower physical activity, and lower daily vegetables/fruits consumption compared to white women (p < 0.001). Non-white women in Brazilian communities are susceptible to increased stress and cardiovascular disease risk, which adds up to disparities in access to the public health system.

## Resumo

Avaliações psicossociais raramente são realizadas com indivíduos residentes na comunidade, especialmente aqueles com maior risco de doença cardiovascular. Este estudo tem como objetivo avaliar o estresse perceptivo e o risco cardiovascular entre mulheres em um grande estudo transversal realizado em comunidades brasileiras. Foram incluídas mulheres com idade superior a 18 anos de 500 unidades básicas de saúde (UBS) públicas do Brasil. Todas as participantes foram submetidas a consulta clínica e aplicação de questionários. Os dados foram utilizados para identificar estilo de vida saudável, tabagismo e autopercepção de estresse psicológico. O índice de risco (NRS) do *National Health and Nutrition Examination Survey* (NHANES) foi utilizado para estimar o risco cardiovascular. As informações de etnia foram autorreferidas, considerando mulheres brancas versus não brancas (negras, pardas e pardas). Um total de 93.605 pacientes foram recrutados em um ambiente de atenção primária, dos quais 62.200 (66,4%) eram mulheres. A autopercepção intensa e grave de estresse foi maior em mulheres não brancas em casa (p < 0,001), no trabalho (p = 0,008), socialmente (p < 0,001) e financeiramente (p < 0,001) em comparação com mulheres brancas. Portanto, a NRS indica que as mulheres não brancas apresentaram maior risco cardiovascular, menor atividade física e menor consumo diário de vegetais/frutas em comparação às mulheres brancas (p < 0,001). As mulheres não brancas nas comunidades brasileiras são suscetíveis ao aumento do estresse e do risco de doenças cardiovasculares, o que aumenta as disparidades no acesso ao sistema público de saúde.

## Resumen

Raramente se realizan evaluaciones psicosociales con personas que viven en la comunidad, especialmente aquellas con mayor riesgo de enfermedad cardiovascular. Este estudio tiene como objetivo evaluar el estrés perceptivo y el riesgo cardiovascular entre las mujeres en un gran estudio transversal realizado en comunidades brasileñas. Se incluyeron mujeres mayores de 18 años de 500 unidades básicas de salud (UBS) públicas de Brasil. Todas las participantes fueron sometidas a una consulta clínica y aplicación de cuestionarios. Los datos se utilizaron para identificar el estilo de vida saludable, el tabaquismo y la autopercepción del estrés psicológico. Se utilizó la puntuación de riesgo (NRS) de la *Encuesta Nacional de Examen de Salud y Nutrición* (NHANES) para estimar el riesgo cardiovascular. La información étnica fue autoinformada, considerando mujeres blancas versus no blancas (negras, marrones y mestizas). Se reclutó a un total de 93.605 pacientes en un entorno de atención primaria, de los cuales 62.200 (66,4%) eran mujeres. La autopercepción intensa y severa del estrés fue mayor entre las mujeres no blancas en el hogar (p < 0,001), en el trabajo (p = 0,008), socialmente (p < 0,001) y financieramente (p < 0,001) en comparación con las mujeres blancas. Por lo tanto, el NRS indica que las mujeres no blancas tenían mayor riesgo cardiovascular, menor actividad física y menor consumo diario de verduras y frutas en comparación con las mujeres blancas (p < 0,001). Las mujeres no blancas en las comunidades brasileñas son susceptibles a un mayor estrés y riesgo de enfermedades cardiovasculares, lo que se suma a las disparidades en el acceso al sistema de salud pública.

## Introduction

Perceived stress represents the psychological perception of environmental demands exceeding individual coping resources and is a core component of the stress process, resulting in adverse physical outcomes, including cardiovascular diseases ^1^. A recent study showed that the combination of high stress symptoms was associated with increased risk of cardiovascular disease in low income countries ^2^.

Psychosocial evaluations are rarely conducted with community dwellers, especially individuals with higher cardiovascular disease risk. Cardiovascular disease is the leading cause of death worldwide and controlling the associated risk factors is a continuous challenge ^3^ ^,^ ^4^. Sociodemographic disparities can be determinant for cardiovascular disease development ^5^. Understanding the difference between sexes on cardiovascular risk factors is important to plan objective strategies to reduce the inequities of the burden of cardiovascular disease ^6^ ^,^ ^7^.

The association of individual cardiovascular disease risk factors and psychological stress is complex, and a simple pattern of causes and effects is not easily distinguished. Few studies have examined the association of psychological stress perception and risk-factors in community-dwelling patients, especially women.

This study aimed to evaluate the psychological stress perception and the cardiovascular risk factors of women assisted in primary care and specialized health services within Brazilian communities. Women, facing challenges such as mental health issues, depression, financial stress, and household stress exhibit distinct susceptibilities to psychosocial factors influencing cardiovascular risk ^8^. Disparities between social classes and race further complicate the understanding of cardiovascular risk among women, with black women experiencing disproportionately higher prevalence of risk factors such as hypertension and obesity ^9^.

Despite the well-established relation between psychosocial variables and cardiovascular risk, the investigation into the connection between cardiovascular risk and psychological stress has been less extensive regarding women. This gap underscores a need for research focused on women, considering their distinct physiological and psychosocial profiles, to elucidate the complex interplay between psychosocial factors and cardiovascular health. By prioritizing women as the focus of the study can facilitate the development of tailored interventions aimed at mitigating cardiovascular risk factors and improving overall cardiovascular health outcomes.

In summary, studying cardiovascular risk in women, with a particular focus on mental health, depression, financial stress, and household stress, is crucial due to the intricate interplay between psychosocial factors and physical health. Understanding the disparities among different social classes and racial groups further emphasizes the importance of targeted research in this area. By addressing the existing gaps in knowledge and exploring the nuanced associations between psychosocial variables and cardiovascular risk in women, we can develop more effective strategies to prevent and manage cardiovascular disease in this population.

## Material and methods

### Participants

This was an observational, cross-sectional study, using a nonrandomized consecutive sampling technique to include subjects of both sexes aged over 18 years, who were evaluated in 500 public basic health units (BHU) in the State of São Paulo, in Brazil. The inclusion criteria were that the subjects should be aged over 18 years, from the general population living near a BHU. Subjects were excluded if they reported not using a given healthcare unit.

### Measures

Data were collected from clinical evaluations, standard questionnaires, face-to-face interviews, and electronic databases ^5^ ^,^ ^10^. The following information was used to classify a healthy lifestyle: daily consumption of fruits, legumes, and vegetables; physical activity (moderate effort at least three times per week); smoking status; and self-perceived psychological stress (social, domestic, financial, and work-related stress). All data were collected before the SARS-CoV2 pandemic.

The *National Health and Nutrition Examination Survey* (NHANES) risk score (NRS) ^11^ was used to estimate the cardiovascular risk among sexes and ethnicity. The ethnic groups were described based on patients’ self-reported ethnicity information and grouped into white and non-white (black, brown and mixed-race) for comparisons.

### Statistics analysis

For practical reasons, a non-probabilistic selection of sites and participants were used. The sample size of 550 patients was sufficient to estimate the prevalence of risk factors with 5% precision and a 95% confidence interval (95%CI). Descriptive statistics were used to estimate the prevalence of cardiovascular risk factors and stress components by sex and ethnicity. The results were analyzed using descriptive statistics and chi-squared test for comparison. Quantitative variables by group, either the Student’s t- or Mann-Whitney tests were used in comparisons of sex and ethnicity. The level of significance adopted was p < 0.05. Data was analyzed using the SPSS version 23.0 (https://www.ibm.com/).

### Ethics statements

The protocol was approved by the local ethics committee (Dante Pazzanese Institute, protocol n. 4,639), and a written informed consent was obtained from all participants prior to data assessment.

## Results

### Perceptual psychological stress related to sex

A total of 93,605 participants were enrolled from a primary care setting, of which 62,200 (66.4%) were female and 31,402 were male. More than 50% of women and 37% of men described significant stressful event on the 12 months prior. Women had reported intense and severe self-perception of domestic (28% vs. 13%; p < 0.001), social (11% vs. 8%; p < 0.001) and financial stress (26% vs. 19%; p < 0.001) compared to men. Self-perceived work-related stress, considering levels of intense and severe stress, were higher in men (15%) than in women (14%), as described in Table 1.

**Table 1:** Clinical characteristics and self-perceived stress of the total population by sex.

	Women	Men	Total	p-value
Age (years) [mean, (SD)]	53.4 (12.1)	54.9 (11.6)	53.9 (11.8)	< 0.001
Systolic blood pressure (mmHg) [mean (SD)]	128 (19)	132 (20)	129 (19)	< 0.001
Diastolic blood pressure (mmHg) [mean (SD)]	80 (11)	83 (12)	81 (12)	< 0.001
BMI (kg/m^2^) [mean (SD)]	28.6 (6.6)	27.5 (4.5)	28.5 (5.3)	< 0.001
Diabetes mellitus [n (%)]	9,500 (15.2)	5,085 (16.2)	14,585 (15.6)	< 0.001
Tobacco [n (%)]				
Never	42,170 (67.7)	16,474 (52.4)	58,644 (62.5)	< 0.001
Current	8,912 (14.3)	5,785 (18.4)	14,697 (15.7)	< 0.001
Former	11,221 (18.0)	9,198 (29.2)	20,419 (21.8)	< 0.001
Lifestyle [n (%)]				
Moderate physical activity	17,490 (28.1)	10,651 (33.9)	28,141 (30.0)	< 0.001
Daily fruit intake	36,644 (58.8)	15,031 (47.8)	51,675 (55.1)	0.001
Daily vegetable or legume intake	40,607 (65.2)	17,469 (55.5)	58,076 (62.0)	0.001
Self-perceived stress [n (%)]				
Important stress event on the 12 months prior	32,124 (51.6)	11,729 (37.3)	43,853 (46.8)	< 0.001
Domestic				
None	9,841 (15.8)	8,275 (26.3)	18,116 (19.3)	< 0.001
Little	14,451 (23.2)	8,804 (28.0)	23,255 (24.8)	< 0.001
Moderate	20,214 (32.5)	10,218 (32.5)	30,432 (32.5)	< 0.001
High or severe	17,742 (28.5)	4,138 (13.2)	21,880 (23.4)	< 0.001
Work-related				
None	33,467 (53.8)	14,758 (46.9)	48,225 (51.5)	< 0.001
Little	8,367 (13.4)	5,374 (17.1)	13,741 (14.7)	< 0.001
Moderate	11,229 (18.0)	6,508 (20.7)	17,737 (18.9)	< 0.001
High or severe	9,198 (14.8)	4,800 (15.3)	13,998 (14.9)	< 0.001
Social				
None	26,996 (43.4)	14,021 (44.6)	41,017 (43.8)	< 0.001
Little	14,511 (23.3)	7,837 (24.9)	22,348 (23.9)	< 0.001
Moderate	13,632 (21.9)	6,943 (22.1)	20,575 (22.0)	< 0.001
High or severe	7,105 (11.4)	2,636 (8.4)	9,741 (10.4)	< 0.001
Financial				
None	12,619 (20.3)	7,723 (24.6)	20,342 (21.7)	< 0.001
Little	13,213 (21.2)	7,283 (23.2)	20,496 (21.9)	< 0.001
Moderate	19,755 (31.7)	10,204 (32.5)	29,959 (32.0)	< 0.001
High or severe	16,668 (26.8)	62,22 (19.8)	22,890 (24.4)	< 0.001

BMI: body mass index; SD: standard deviation.

### Perceptual psychological stress related to women’s ethnicity

The evaluation based on women’s ethnicity showed that the non-white group (25,562; 41%) had higher prevalence of hypertension (p < 0.001), practiced less moderate physical activity (p < 0.001), and consumed less fruits, legumes, and vegetables (p < 0.001, for all comparisons). Intense and severe self-perceived stress was higher within non-white women at home (p < 0.001), at work (p = 0.008), socially (p < 0.001) and financially (p < 0.001) compared to white women. Furthermore, current and former smokers were more prevalent among non-white women (p < 0.001), as shown in Table 2.

**Table 2:** Clinical characteristics and self-perception of stress by ethnicity in 62,200 women in Brazilian communities.

Variables	White women (n = 36,638)	Non-white women * (n = 25,562)	All women (n = 62,200)	p-value
Age (years) [mean (SD)]	54.1 (11.8)	52.3 (11.3)	53.4 (12.1)	< 0.001
Systolic blood pressure (mmHg) [mean (SD)]	127 (19.0)	128 (19.0)	128 (19.0)	< 0.001
Diastolic blood pressure (mmHg) [mean (SD)]	80 (11.0)	81 (12.0)	80 (11.0)	< 0.001
BMI (kg/m^2^) [mean (SD)]	28.4 (5.5)	28.8 (5.7)	28.6 (6.6)	< 0.001
Diabetes mellitus [n (%)]	5,478 (14.9)	4,022 (15.7)	9,500 (15.2)	0.007
Hypertension [n (%)]	18,383 (50.1)	14,225 (55.6)	32,608 (52.3)	< 0.001
Tobacco [n (%)]				
Never	25,681 (70.0)	16,476 (64.4)	42,157 (67.7)	< 0.001
Current	4,792 (13.1)	4,119 (16.1)	8,911 (14.3)	< 0.001
Former	6,218 (16.9)	5,001 (19.5)	11,219 (18.0)	< 0.001
Lifestyle [n (%)]				
Moderate physical activity	10,804 (29.5)	6,680 (26.1)	17,484 (28.1)	< 0.001
Daily fruit intake	22,632 (61.7)	14,003 (54.7)	36,635 (58.8)	< 0.001
Daily vegetable or legumes intake	24,541 (66.9)	16,061 (62.8)	40,602 (65.2)	< 0.001
Self-perceived stress [n (%)]				
Important stress event on the 12 months prior	18,769 (51.2)	13,349 (52.2)	32,118 (51.6)	0.014
Domestic				
None	5,880 (16.0)	3,955 (15.5)	9,835 (15.8)	< 0.001
Little	8,754 (23.9)	5,694 (22.3)	14,448 (23.2)	< 0.001
Moderate	11,981 (32.7)	8,232 (32.2)	20,213 (32.5)	< 0.001
High or severe	10,045 (24.7)	7,695 (30.0)	17,740 (28.6)	< 0.001
Work-related				
None	19,586 (53.4)	13,874 (54.2)	33,460 (53.8)	< 0.001
Little	4,918 (13.4)	3,446 (13.5)	8,364 (13.4)	< 0.001
Moderate	6,695 (18.3)	4,532 (17.7)	11,227 (18.0)	< 0.001
High or severe	5,466 (14.9)	3,730 (14.6)	9,196 (14.5)	< 0.001
Social				
None	15,724 (42.9)	11,268 (44.0)	26,992 (43.4)	< 0.001
Little	8,674 (23.7)	5,834 (22.8)	14,508 (23.3)	< 0.001
Moderate	8,172 (22.3)	5,460 (21.3)	13,632 (21.9)	< 0.001
High or severe	4,085 (11.1)	3,019 (11.8)	7,104 (11.4)	< 0.001
Financial				
None	7,688 (21.0)	4,928 (19.3)	12,616 (20.3)	< 0.001
Little	7,908 (21.6)	5,302 (20.7)	13,210 (21.2)	< 0.001
Moderate	11,734 (32.0)	8,018 (31.3)	19,752 (31.7)	< 0.001
High or severe	9,337 (25.4)	7,329 (28.7)	16,666 (26.8)	< 0.001

BMI: body mass index; SD: standard deviation.

* Non-white women = black, brown and mixed-race.

### Cardiovascular risk among women’s ethnicity

**Figure 1:**
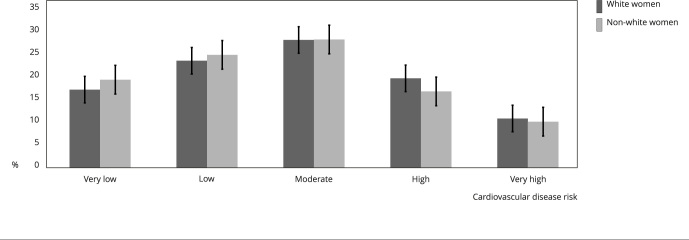
The *National Health and Nutrition Examination Survey* (NHANES) cardiovascular disease risk comparison among women ethnicity in Brazilian community setting.

The NRS indicates that non-white women had a higher cardiovascular risk compared to white women (p < 0.001) (Figure 1). Hypertension was the most prevalent cardiovascular risk factor for women. Conversely, there were less cases of diabetes mellitus (p < 0.001) and former smokers (p < 0.001) among women. After calculating the NRS, most women had moderate or lower risk, and almost 30% had high or very high risk (p < 0.001) as is described in Table 3.

**Table 3:** The *National Health and Nutrition Examination Survey* (NHANES) risk score (NRS) for cardiovascular disease for women and men on a community setting.

NRS	Women		Men		Total		p-value
	n	%	n	%	n	%	
Risk < 5%: very low	10,758	18.2	3,623	12.3	14,381	16.2	< 0.0001
Risk 5-10%: low	14,301	24.2	6,101	20.7	20,402	23.0	< 0.0001
Risk 10-20%: moderate	16,757	28.4	7,071	23.9	23,828	26.9	< 0.0001
Risk 20-30%: high	10,982	18.6	6,289	21.3	17,271	19.5	< 0.0001
Risk > 30%: very high	6,285	10.6	6,460	21.9	12,745	14.4	< 0.0001

## Discussion

Our study presents women’s high prevalence of perceived stress in community settings and a low cardiovascular risk evaluated by NRS when compared to men. However, our findings are more evident when considering ethnicity in the women group. Stress and depression are strong cardiovascular risk factors in Latin America, increasing the chances of a stroke by up to seven times, as well as constitute important causes of disability and death in the region ^12^. In Brazilian communities, non-white women are susceptible to increased perceptual stress, which may affect cardiovascular outcomes ^13^ ^,^ ^14^ ^,^ ^15^, which in turn increases disparities to the access to public healthcare system.

Public healthcare strategies based on data of mental, social, and physiological components are necessary for risk reduction of the population. Therefore, social support is a multidimensional construct, with emotional support viewed as more nurturing than either informational or tangible support. Emotional support also has stronger cardioprotective effects, especially in subjects with elevated psychological stress ^9^ ^,^ ^16^.

Health disparities, differences in the incidence, mortality and burden of diseases, and other adverse health conditions found between specific populations are worsened in low income countries ^17^.

There are several benefits from using the NRS to estimate cardiovascular risk in community settings. Firstly, it provides a standardized and validated tool for assessing cardiovascular risk, allowing for consistent risk assessment across different populations and settings. Secondly, the NRS incorporates a comprehensive range of risk factors, including age, gender, ethnicity, blood pressure, cholesterol levels, smoking status, and diabetes status, providing a more holistic assessment of cardiovascular risk, when compared to individual risk factors. Additionally, the NRS has been shown to accurately predict cardiovascular events and mortality, making it a valuable tool for identifying individuals at high risk who may benefit from targeted interventions ^18^.

Our findings reveal variations in the impact of risk factors among women of different ethnicities, even within the same community. Specifically, the NRS indicated a higher prevalence of high and very high cardiovascular risk among white women compared to non-white women. Such observation agrees with the *Prospective Urban Rural Epidemiology* (PURE) study, which investigated cardiovascular disease incidence among income strata in various countries. Despite a higher prevalence of risk factors in high income countries, mortality rates were disproportionately higher in very low and low income countries, suggesting the presence of more severe or poorly managed diseases - a phenomenon called the paradox of cardiovascular disease in the world ^17^.

Regarding the prevalence of self-perceived stress between sexes, women are more affected than men. Notably, no statistical difference between white and non-white women was found, in both groups more than 50% of the women experienced important stress on the 12 months prior to the study. The Latin America is deeply affected by socioeconomic inequalities. Women represents a significant part of the economic effort of families, as they are responsible for most of the household chores, while receiving lower wages, and facing more obstacles to participate in the formal labor market ^19^. All these factors could explain the cause to women frequently reporting more stress and depression rates than men ^7^ ^,^ ^20^.

Non-white women reported feeling more financially stressed than other women. Those women are constantly seeking higher social acceptance and are directly impacted by structural racism, exposing them to severe stress. It is noteworthy that black women have even worse salaries and positions among women, regardless of social status ^21^ ^,^ ^22^.

The structural racism exposes non-white women to higher levels of stress, which can significantly impact their cardiovascular health. Studies have demonstrated that chronic stress resulting from racial discrimination can lead to adverse health outcomes, including cardiovascular diseases ^23^. Additionally, unequal access to quality healthcare contributes to a heavier burden of cardiovascular diseases among non-white women in low income countries ^18^.

Understanding these disparities is crucial for informing intervention strategies and health policies aimed at reducing cardiovascular risk in these vulnerable populations. Health equity promotion requires multifaceted approaches that address not only traditional risk factors but also the underlying social and racial inequalities contributing to health disparities.

The control of cardiovascular disease risk factors may help to control psychological symptoms, as evinced by recent data showing that the use of beta-blockers for improvement of hypertension control and heart failure may modulate mental stress response by inhibiting sympathetic nervous system activity ^16^. Our study did not measure medication uses, but we have verified that 50% of women have high blood pressure. We believe that, like beta-blockers, other medications with pleiotropic effects can help to reduce psychological risk, as a cardiovascular outcome in individuals living in communities.

The long-term health impact of self-perceived stress in global health and cardiovascular outcomes reserves further investigations in the future. Therefore, ecological factors not measured in this study may increase the risk of psychological alterations in women that have an elevated perceptual stress, such as the recent COVID-19 pandemic.

Age is an independent risk factor for cardiovascular disease in adults, but this risk is compounded by additional factors, including frailty, obesity, and diabetes. These factors are known to complicate and enhance cardiac risk factors usually associated with aging ^24^.

Our sample has a high prevalence of diabetes but show expected values for the age, for both sexes. However, it is important to highlight that diabetes alone increases cardiovascular risk by up to 2.5 times ^24^ ^,^ ^25^. Additionally, the coexistence of depression in individuals with diabetes further exacerbates cardiovascular risk, as depression has been shown to independently contribute to a higher incidence of cardiovascular events ^26^.

Worldwide, tobacco use increases the chance of stroke or acute myocardial infarction by 2 times ^2^. Brazil had implemented 15 years ago a national policy to reduce its consumption and have reduced , reduced tobacco use from 15% to 9%, one of the best outcomes of Latin America ^27^ ^,^ ^28^. These data shows that specific groups still have a high prevalence of tobacco use and deserve attention from health authorities.

### Strengths and limitations

By identifying important gaps in cardiovascular disease risk factors and elevated self-perceived stress, this study demonstrates considerable potential to improve mental health care and cardiovascular prevention in communities settings. Although our data reflect real psychological perceptual condition of non-white woman in a representative cohort of high risk patients who qualify for primary care according to the primordial preventive concept.

Our study has several limitations. Although we evaluated nonrandomized basic health units in the State of São Paulo, including all users of the units, we did not identify the quantity of prescribed medications, whether it was dispensed or if the patients complied with therapy for all evaluated clinical conditions. Our study make no assumptions on medical compliance, which is often low in chronic conditions, especially among community-dwelling individuals. Therefore, the data reported in our study likely described a real-world management and perception scenario of primary prevention in Brazil. We used NRS, because over half of our patients did not have documented lipid and glucose profile. Patients in primary prevention without documented laboratory results typically had high-risk characteristics, and psychological factors may explain those conditions.

Notably, the data for this study were obtained from patients assisted in Brazilian BHU, in which healthcare is costless, even prescription drugs are free at specific locations. These factors should be considered when comparing our findings to other healthcare systems, in which discrepancies in quality care may be expected in private healthcare users and reference health centers.

## Conclusion

Non-white women in Brazilian communities are susceptible to increased stress and unhealthy lifestyle, which in turn adds up to disparities in the access to public health system. Our result urges the implementation of psychosocial actions to improve women’s mental health, especially the non-whites who live in communities.
